# Another Word about Christian Science

**Published:** 1902-10

**Authors:** 


### *EDITORIAL NOTES AND COMMENTS.*

The Business office of THE JOURNAL-RECORD is No. 64 Marietta Street.

The Editorial office is 318-319-320 The Prudential.

Address all Business communications to Dr. M. B. Hutchins, Mgr.

Make remittances payable to THE ATLANTA JOURNAL-RECORD OF MEDICINE.

On matters pertaining to the Editorial and Original Communications address Dr. Bernard Wolff, Atlanta.

Reprints of original articles will be furnished at cost price. Requests for the same should always be made *on the manuscript*.

We will present, post-paid, on request, to each contributor of an original article, twenty (20) marked copies of THE JOURNAL-RECORD containing such article.

### ANOTHER WORD ABOUT CHRISTIAN SCIENCE.

THE alleged steady increase in the disciples of the delectable cult of "Christian Science" fills one with amazement and brings the conviction that the fools in the world outnumber the philosophers in overwhelming ratio. Belief in the absurd and monstrous doctrine promulgated by the ancient sybil of Lynn, Mass., is simply a matter of determining the amount of nonsense and incoherent gibberish that the mind of man can take up before it melts into nothing like a lump of dirt. The vagaries of this superstition have afforded much amusement to that part of the public which enjoys a modicum of common sense; they have supplied the theme for psychologic analysis and have been the object of objurgatory criticisms and frank challenges from the medical profession. But like all other rank and noisome growths, plucking up seems but to stimulate that which remains overlooked, and in spite of opposition and division they continue to grow and defile with their mephitic vapors the nostrils of sense and reason.

It would be very far from true to assert that all who believe in this astonishing doctrine are fools, but it is well within reason to question their mental balance. Belief, to be taken from the category of open-mouthed credulity, must be based upon proven facts. Otherwise it is entitled to no consideration. Those who are prepared to accept the non-existence of matter cannot deny the possibility of occurrence of any phenomenon which notoriously goes contrary to the established order of nature. This would be a very unsettled state of things indeed, and we would be cast back into that primitive chaos from which we were so tediously extricated.

A singular phase of this agnosticism toward Nature and her prescribed modes of action is the greed and commercialism which are spun into the fabric of the "scientist." The prime purpose of churches of the cult is to promulgate the "faith" and to sell the literature of it. At least this is the view taken by a Philadelphia judge in denying a charter for such an institution.

We are told that salvation is free, but in this assertion the item of pew-rent and sundry copies of "Science and Health" are plainly overlooked. It was for such an offense that He whose name is blasphemously attached to this modern heresy routed out the desecrators of a sacred edifice, and blasted them with the heat of His wrath. If the proceeds from the sale of the dope dreams that parade as literature were devoted to the alleviation of human suffering, to the raising the smallest fraction of the down-trodden and afflicted, there might be some extenuation for the inharmonious combination of commercialism and "religion." But if one penny has ever been devoted to eleemosynary or philanthropic purposes it does not appear in the balance sheet. The money has doubtless gone to clothing the immaterial forms and lining the incorporate bellies of the devout, and they are as sleek as priests and do not purchase at smoke sales.

As a religion it belongs to the prescientific era when charms and relics and amulets were used as fetishes to prevent and cure disease.

If it were possible to systematize from the jumble of statements and vague premises contained in "Science and Health," the system would be an anachronism in the continuity of religious development. As a science, which is, as Tyndall calls it, the logic of nature, certainly the cheap and trumpery theories can lay no mite of a claim to it.

We shall probably live to see the extermination of this silly and idle sect by a process of self-immolation, but we may expect in place of it another equally as offensive and preposterous. There will always be the emotional superficial type of man who will seek, as they have sought, after a sign.

